# ATG8f Interacts with Chilli Veinal Mottle Virus 6K2 Protein to Limit Virus Infection

**DOI:** 10.3390/v15122324

**Published:** 2023-11-26

**Authors:** Chenglong Ji, Jingya Zhou, Daoyong Yang, Bowen Yuan, Rongxia Tang, Yong Liu, Dehui Xi

**Affiliations:** 1 Key Laboratory of Bio-Resource and Eco-Environment of Ministry of Education, College of Life Sciences, Sichuan University, Chengdu 610065, China; jclhaokuo@sina.com (C.J.);; 2 Institute of Plant Protection, Sichuan Academy of Agricultural Sciences, Chengdu 610066, China

**Keywords:** autophagy, ChiVMV, ATG8f, 6K2

## Abstract

Autophagy, as a conserved protein degradation pathway in plants, has also been reported to be intricately associated with antiviral defense mechanisms. However, the relationship between chilli veinal mottle virus (ChiVMV) and autophagy has not been investigated in the existing research. Here, we reveal that ChiVMV infection caused the accumulation of autophagosomes in infected *Nicotiana benthamiana* leaves and the upregulation of autophagy-related genes (*ATGs*). Moreover, the changes in gene expression were correlated with the development of symptoms. Treatment with autophagy inhibitors (3-MA or E-64D) could increase the infection sites and facilitate virus infection, whereas treatment with the autophagy activator (Rapamycin) limited virus infection. Then, ATG8f was identified to interact with ChiVMV 6K2 protein directly in vitro and in vivo. The silencing of *ATG8f* promoted virus infection, whereas the overexpression of *ATG8f* inhibited virus infection. Furthermore, the expression of 6K2-GFP in *ATG8f-* or *ATG7-*silenced plants was significantly higher than that in control plants. Rapamycin treatment reduced the accumulation of 6K2-GFP in plant cells, whereas treatment with the inhibitor of the ubiquitin pathway (MG132), 3-MA, or E-64D displayed little impact on the accumulation of 6K2-GFP. Thus, our results demonstrated that ATG8f interacts with the ChiVMV 6K2 protein, promoting the degradation of 6K2 through the autophagy pathway.

## 1. Introduction

Autophagy is a conserved intracellular degradation pathway that degrades and recycles functionally impaired organelles or other cytoplasmic components through vesicular structures (vacuoles in plants and yeast, and lysosomes in mammals) [1,2]. Autophagy in eukaryotes can be divided into chaperone-mediated autophagy (CMA), macroautophagy, and microautophagy [3,4]. Macroautophagy is the formation of a unique double-membrane structure (autophagosome) from the endoplasmic reticulum under external stimulation, which encapsulates the autophagic cargo into lysosomes or vacuoles for degradation [5]. The commonly described autophagy represents macroautophagy. More than 40 *ATGs* have been identified [6], and it is generally accepted that the autophagy process mainly involves the induction, nucleation, and expansion of the phagophore; maturation of the autophagosome; and fusion with the vacuole or lysosome for substrate degradation [7,8,9,10,11].

Autophagy is a key mechanism for the maintenance of homeostasis in plant cells, as it provides energy and circulating nutrients to sustain survival under starvation stress [12,13]. Other abiotic stresses, including salt stress [14,15], drought stress, and hypoxia stress [16], can also induce autophagy [16,17]. In addition to abiotic stresses, infection by pathogenic microorganisms, including viruses, can also activate autophagy, as in the case of the necrotic pathogenic fungus *Botrytis cinerea* after it invaded *Arabidopsis thaliana*. The signal pathway was mediated by jasmonic acid (JA) and *WRKY33*-upregulated autophagy genes and promoted the formation of autophagosomes [18]. The effector protein PexRD54 of *Phytophthora infestans* blocks the autophagic clearance of plant or pathogen proteins that negatively affect immunity by interacting with ATG8 to enhance autophagosome assembly [19].

ATG8 is a key adaptor protein in autophagy, and it was first identified as an autophagy protein in the early 1990s [20]. ATG8 is exposed to c-terminal glycine after treatment with ATG4 cysteine protease. The mature ATG8 is bound to the membrane lipid phosphatidylethanolamine, and the bound ATG8 connects the autophagy adapter and receptor protein to the expanding phagophore [21,22]. Some viral proteins and plant proteins had been reported to interact directly or indirectly with ATG8 family proteins in plants [23,24,25,26,27]. For instance, the βC1 of cotton leaf curl Multan virus (CLCuMuV) interacted with ATG8f and was degraded by autophagy [23], the NbP3IP directed the degradation of rice stripe virus p3 protein through interaction with the autophagy-related protein NbATG8 to limit virus infection [26], and the NIb encoded by the turnip mosaic virus (TuMV) interacted with Beclin1 to limit viral infection through Atg8a-targeted autophagic degradation [24].

*Potyvirus* is the largest genus of plant viruses with a positive single-stranded RNA genome [28]. *Chilli veinal mottle virus* belongs to the genus *Potyvirus* of the family *Potyviridae*, which encodes one polyprotein and hydrolyzes into 11 mature viral proteins, including 6K2, NIb, HC-Pro, Vpg, etc. The membrane-bound protein 6K2 can generate the formation of endoplasmic reticulum (ER)-derived vesicles for intercellular movement of potyviral replication complexes (VRCs) and viral RNA replication [29,30,31], and it is critical for intercellular movement and systemic infection of potyviruses. NIb is a viral RNA-dependent RNA polymerase (RdRp). HCPro and Vpg are two known RNA-silencing viral suppressors (VSRs) [32]. In addition, NIb, HCPro, and VPg, when encoded by other viruses in the genus *Potyvirus*, have been reported to interact with autophagic components [24,33,34].

In recent years, the outbreak of ChiVMV has caused great economic losses to the tobacco industry of China and has severely restricted the production of tobacco [35]. It is also a serious threat to solanaceous plants worldwide [36,37]. In this study, we demonstrated that ChiVMV infection caused the specific expression of *ATGs* and accumulation of autophagosomes in tobacco plants. Activation of autophagy promoted plants’ resistance to ChiVMV, and ATG8f specifically interacted with the ChiVMV 6K2 protein, promoting 6K2 degradation through the autophagy pathway.

## 2. Materials and Methods

### 2.1. Plant Materials and Virus Inoculation

Wild-type *N. benthamiana* plants were grown in a greenhouse with a 12 h light/12 h dark cycle (100 μM m^−2^ s^−1^) at 23–26 °C. Six-week-old seedlings were mechanically inoculated with ChiVMV isolate Yp8 (GenBank: KC711055.1), and leaves inoculated with phosphate-buffered saline (50 mM PBS, pH 6.8) were used as the mock treatment. The virus source of inoculation was the same batch of virus-carrying leaf homogenates which were prepared uniformly and stored in a −80 °C ultralow-temperature refrigerator.

### 2.2. Vector Construction and Agrobacterium Infiltration

The pTRV1, pTRV2, and TRV: PDS vectors used for virus-induced gene silencing (VIGS), were prepared as described by Zhu et al. [38]. For the silencing of *NbATG3* (GenBank: KX369396.1), *NbATG7* (GenBank: KX369398.1), and *NbATG8f* (GenBank: KU561372.1), cDNA fragments of the *NbATG3*, *NbATG7*, and *NbATG8f* sequences were amplified by reverse-transcription PCR (RT-PCR) with specific primers and inserted into the pTRV2 vector to generate the TRV: NbATG3, TRV: ATG7, and TRV: ATG8f plasmid driven by the CaMV 35S promoter. Next, the pTRV1, TRV: NbATG3, TRV: ATG7, and TRV: ATG8f vectors were transferred separately into different *Agrobacterium tumefaciens* strain GV3101, and then positive colonies were infiltrated into *N. benthamiana* plants, as previously reported by Zhu et al. [38]. For the overexpression of *NbATG8f* and *6K2*, the coding sequence (CDS) of *NbATG8f* and *6K2* was amplified by RT-PCR with specific primers and inserted into the pCM1307 (FLAG), pCAMBIA1300-eGFP (GFP), and pPGDR (RFP) vector to generate the ATG8f-GFP, ATG8f-flag, 6K2-GFP, and 6K2-RFP plasmid, respectively. All primers used for vector construction are listed in Supplementary Table S1.

### 2.3. Transmission Electron Microscopy (TEM)

The fresh samples were prefixed in a mixed solution of 3% glutaraldehyde, followed by post-fixing in 1% osmium tetroxide, dehydration in a series of acetone, and infiltration in Epon812; then, they were finally embedded. The semi-thin sections were stained with methylene blue, and ultrathin sections were cut with a Mond knife and stained with uranyl acetate and lead citrate. The sections were examined using a transmission electron microscope (TEM; HITACHI, H-600IV, Tokyo, Japan) [39].

### 2.4. RNA Extraction and Quantitative RT-PCR (qRT-PCR)

Total RNA was isolated from leaves, using TRIzol reagent (Invitrogen, Carlsbad, CA, USA), followed by the removal of genomic DNA with DNase treatment. Reverse transcriptase was used to obtain cDNA for qPCR. Quantitative real-time PCR was performed on Bio-Rad iCycler (Bio-Rad Laboratories, Hercules, CA, USA), using HIEFF qPCR SYBR Green Master Mix (Yeasen, Shanghai, China). The expression level of *N. benthamiana Actin* (*NbActin,* GenBank: AY179605.1) was detected and used for standardization. All experiments were performed three times, and three independent biological replications were performed [40]. The primers used for qRT-PCR analysis are listed in Supplementary Table S1.

### 2.5. Protein Extraction and Western Blot Analysis

The method of extracting plant protein was based on Li et al. [41]. Proteins were detected using anti-ChiVMV coat protein (CP) monoclonal primary antibody (1:5000) and alkaline phosphatase conjugate goat anti-rabbit IgG (1:5000) secondary antibodies. Enhanced blotting signals were detected using PIERCETM ECL Western blotting substrate (Thermo Fisher Scientific, Waltham, MA, USA). Coomassie brilliant blue (CBB) staining of the Rubisco large subunit was used as a loading control [40].

### 2.6. Chemical Treatments

Chemical treatments were performed post 24 h of virus inoculation. Rapamycin was used as an autophagy activator (mammalian target of rapamycin inhibitor) at a concentration of 1 μM. Then, 3-methyladenine (a representative autophagy inhibitor, 3-MA) and proteinase inhibitor E 64 (a lysosomal enzyme inhibitor, E-64D) were used as autophagy inhibitors at a concentration of 5 mM and 100 μM, respectively. MG132 was used as a proteasome inhibitor at a concentration of 100 μM. For the method of diluent configuration, we referred to Huang et al. [42]. Because the reagents were dissolved in dimethyl sulfoxide (DMSO) before dilution, the DMSO solution was used as a control treatment. The prepared solutions were infiltrated into the virus-inoculated leaves, respectively.

### 2.7. Yeast Two-Hybrid (Y2H) Assay

The CDS of *NbATG3*, *NbATG7*, *NbNBR1* (GenBank: MG710800.1), *NbATG8a* (GenBank: KX120976.1), and *NbATG8f* were cloned into pGADT7 to generate the AD vectors, respectively. Likewise, the CDS of *VPg*, *P1*, *HCPro*, *P3N-PINO*, *P3*, *6K1*, *CI*, *6K2*, *NIa*, *NIb,* and *CP* were cloned into pGBKT7 to generate the BD vectors, respectively. The resulting constructs were transferred into yeast strain AH109, and positive colonies were selected and cultured on double dropout (DO) supplement (SD-Leu/-Trp) for 2–3 days. Then, co-transformants were shifted onto quadruple DO supplement (SD-Leu/-Trp/-His/-Ade) to test for possible interactions.

### 2.8. Bimolecular Fluorescence Complementation (BiFC) Assay

The CDS of *NbATG8f* and *6K2* were inserted into the PXY103-nYFP and PXY104-cYFP vector to generate the nYFP-ATG8f and cYFP-6K2 plasmid, respectively. The plasmids were separately transferred into *Agrobacterium tumefaciens* strain GV3101 and co-infiltrated into 6-week-old *N. benthamiana* plants. Positive colonies were selected and cultured at 28 °C for 24 h, and then the bacteria were centrifuged and resuspended in infiltration medium (10 mM MES, pH 5.6, 10 mM MgCl_2_, and 150 μM acetosyringone) to a final OD_600_ of 0.8 for the transformation of *N. benthamiana*. The agroinfiltrated plants were grown in a greenhouse for at least 36 h, and fluorescent signals were observed via a Leica DMIRBE confocal laser scanning microscope (Leica Microsystems, Heidelberg, Germany) [36].

### 2.9. Co-Immunoprecipitation (Co-IP) Assay

The recombinant plasmids 6K2-GFP and ATG8f-flag were separately transferred into *A. tumefaciens* strain GV3101 and co-infiltrated into 6-week-old *N. benthamiana* plants. After being cultivated for 60 h in the greenhouse, these agroinfiltrated leaves were then ground in protein extraction buffer (50 mM HEPES, pH 7.4, 150 mM KCl, 1 mM EDTA, 0.1% Triton X-100, 1 mM DTT, and 1× protease inhibitor cocktail). After centrifugation at 12,000× *g* for 10 min at 4 °C, the supernatant was incubated with anti-GFP agarose affinity gel beads at 4 °C for 3 h. Subsequently, the beads were washed several times, using extraction buffer (20 mM HEPES, pH 7.4, 3 mM MgCl_2_, 50 mM NaCl, 0.1 mM EDTA, and 0.05% Triton X-100), and the adsorbed proteins were eluted from the beads by boiling in 2× SDS loading buffer. After that, the pulled-down proteins were separated by SDS-PAGE and hybridized with anti-flag and anti-GFP antibodies to evaluate the protein interaction.

### 2.10. Statistical Analysis

Samples were analyzed in triplicate, and the data are expressed as the mean ± SD, unless noted otherwise. Statistical significance was determined using one-way ANOVA (Duncan multiple comparisons) or Student’s *t*-test. A difference at *p* < 0.05 was considered significant.

## 3. Results

### 3.1. ChiVMV Infection Activates Autophagy in N. benthamiana

**Figure 1:**
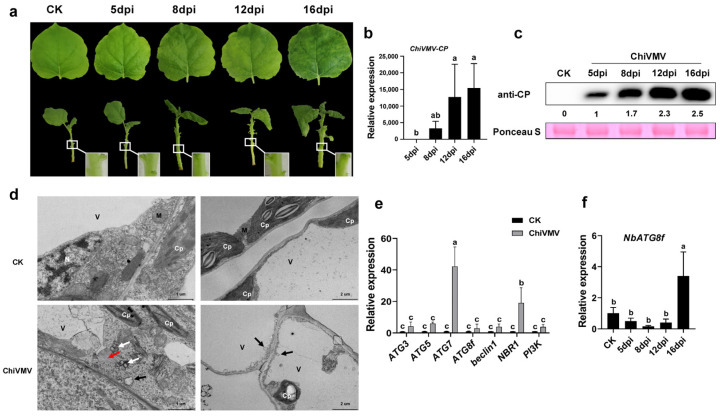
Virus symptoms and autophagy responses in *N. benthamiana* after ChiVMV inoculation. (**a**) Systemic leaf symptoms of *N. benthamiana* at 5 dpi, 8 dpi, 12 dpi, and 16 dpi, respectively. (**b**) Relative expression of viral *CP* gene in *N. benthamiana* leaves at 5 dpi, 8 dpi, 12 dpi, and 16 dpi, respectively. (**c**) Accumulation of viral coat protein (CP) in the systemic leaves. (**d**) Observation of autophagosomes in *N. benthamiana* at 13 dpi. The samples were treated with phosphoric acid buffer solution as the control (CK). The black arrow indicates autophagosomes in plant cells. The white arrow indicates virus inclusions, and the red arrow indicates linear virus. Cp, chloroplast; M, mitochondria; V, vacuole; and N, nucleus. (**e**) Relative expressions of *ATGs* in the systemic leaves at 9 dpi. (**f**) Relative expression of *NbATG8f* at different time points after ChiVMV infection. Data are shown as means ± SD (*n* = 3). Lower case letters indicate statistically significant differences (*p* < 0.05).

To understand the response of *N. benthamiana* to ChiVMV infection, stems and systemically infected leaves were observed and collected at 5 days post-inoculation (dpi), 8 dpi, 12 dpi, and 16 dpi, respectively. The results showed that there was obvious leaf shrinkage and vein clearing in ChiVMV-infected plants at 8 dpi, and the whole leaves turned dark green at 16 dpi (Figure 1a). The relative expression of the viral *CP* gene and accumulation of viral coat protein in systemically infected leaves increased gradually, and this result was consistent with the development of viral infection symptoms (Figure 1b,c). TEM was performed to observe the damage on virus-infected leaf tissues caused by ChiVMV. The result showed that virus inclusions (white arrow) and linear virus (red arrow) were observed in ChiVMV-infected cells (Figure 1d, the left image of the lower panel). More importantly, there existed autophagosomes (black arrow) densely distributed at the inner edge of the vacuole in virus-infected cells (Figure 1d, the right image of the lower panel). Because of the large number of autophagosomes observed in virus-inoculated leaf tissues, the relative expressions of *ATGs*, including *ATG3*, *ATG5*, *ATG7*, *ATG8f*, *Beclin1*, *NBR1*, and *PI3K*, were examined by qRT-PCR. The results showed that *ATG7* and *NBR1* were highly expressed at 9 dpi (Figure 1e). The relative expression of *ATG8f* was also examined at 5 dpi, 8 dpi, 12 dpi, and 16 dpi (Figure 1f), and a strong response was found at the late stage of infection (16 dpi). The above results showed that ChiVMV infection activated autophagy in *N. benthamiana*.

### 3.2. Autophagy Plays a Positive Role in the Antiviral Process of N. benthamiana

**Figure 2:**
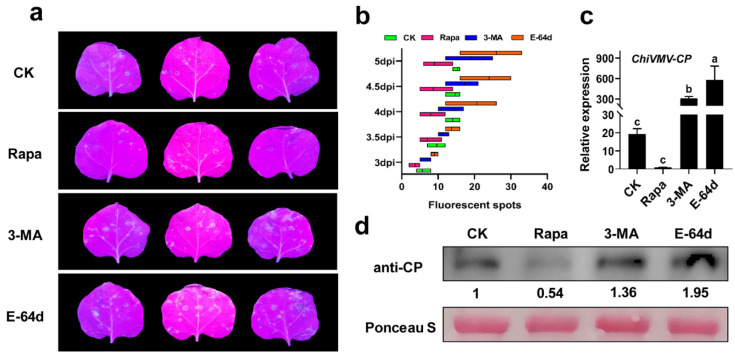
Effect of chemicals treatment on ChiVMV infected *N. benthamiana.* (**a**) Symptom on ChiVMV-GFP-infected *N. benthamiana* leaves treated with Rapa, 3-MA, and E-64d, respectively. (**b**) The number of fluorescent spots on inoculated leaves. (**c**) Relative expressions of viral *CP* corresponding to leaves in (**a**). (**d**) The accumulation of viral coat protein corresponding to leaves in (**a**), and the number represents the relative accumulation. Data are shown as means ± SD (*n* = 3). Lower case letters indicate statistically significant differences (*p* < 0.05).

To investigate the role of autophagy during ChiVMV infection, an autophagy activator (Rapamycin) and inhibitors (3-MA and E-64d) were used in virus-infected *N. benthamiana*. The results showed that there were fewer viral fluorescent spots on Rapamycin-treated plants than on DMSO-treated plants, whereas there were more viral fluorescent spots on 3-MA- or E-64D-treated plants than there were on DMSO-treated plants (Figure 2a,b). The relative expression of viral *CP* and the accumulation of viral coat protein in inoculated leaves were examined at 5 dpi. The results showed that the 3-MA- or E-64D-treated plants showed a higher expression of viral *CP* and a greater accumulation of viral coat protein, whereas the Rapamycin-treated plants exhibited the opposite results (Figure 2c,d). *ATG3* and *ATG7* were known as the key genes regulating autophagy [23]. VIGS was used to silence *ATG3* and *ATG7* in *N. benthamiana* to detect the effect of autophagy on the plant response to ChiVMV infection. The results showed that *ATG3/ATG7*-silenced plants displayed more fluorescent spots on inoculated leaves and stronger fluorescence intensity in systemically infected leaves compared to that in TRV: GUS plants at 5 dpi (Supplementary Figure S1a,b). Accordingly, the results of the qPCR and Western blot analysis showed that the expression of viral *CP* and the accumulation of viral coat protein were higher in *ATG3/ATG7*-silenced plants compared to that in TRV: GUS plants (Supplementary Figure S1d–f). The above results proposed that autophagy played a positive role in the response of *N. benthamiana* to ChiVMV infection.

### 3.3. ATG8f Interacts with ChiVMV-6K2 In Vitro and In Vivo

**Figure 3:**
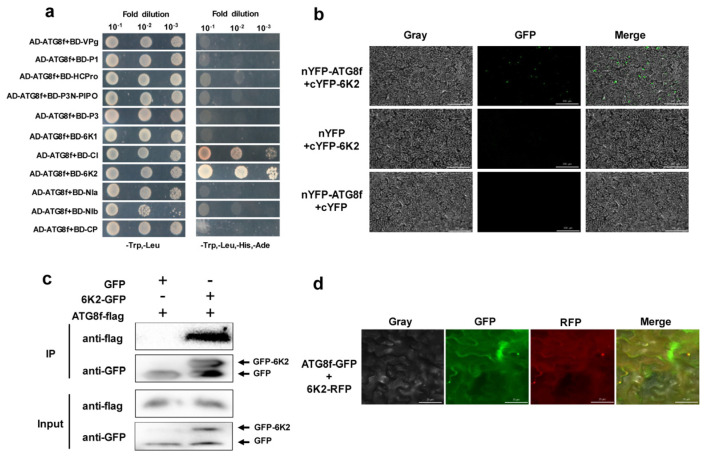
The interaction between ChiVMV-6K2 and ATG8f in vitro and vivo. (**a**) Y2H screening of the interaction between NbATG8f- and ChiVMV-encoded proteins. (**b**) BiFC assays. The green fluorescence indicates an interaction between ATG8f and 6K2. Bars = 200 μm. (**c**) Co-IP assays. GFP-6K2 was co-expressed with FLAG or FLAG-ATG8f in *N. benthamiana* plants. After immunoprecipitation with FLAG beads, the proteins were detected via a Western blot analysis with an anti-FLAG or anti-GFP antibody, respectively. (**d**) Subcellular localization assays of ATG8f and 6K2. Bars = 20 μm. ATG8f-GFP and 6K2-RFP were incorporated into *N. benthamiana* leaves. GFP corresponds to the position of ATG8f, and RFP corresponds to the position of 6K2.

Previous studies reported that ATG8a and ATG8f were directly involved in autophagy-mediated degradation, and NBR1 was an autophagy cargo receptor protein [21,22,43]. Therefore, the Y2H assay was used to screen the possible interactions between four autophagy-related proteins (ATG8a, ATG8f, NBR1, and ATG7) and the eleven proteins encoded by ChiVMV. The results of the Y2H assay showed that only ATG8f interacted with the 6K2 and cylindrical inclusion protein (CI) in yeast cells (Figure 3a and Supplementary Figure S2a–c). Subsequently, the interaction between ATG8f and 6K2 was further investigated. The results of the BiFC assay confirmed that there existed an interaction between nYFP-ATG8f and cYFP-6K2 in plant cells (Figure 3b). Furthermore, fusion protein 6K2-GFP and ATG8f-flag were co-infiltrated into *N. benthamiana* leaves, and flag protein were detected after Co-IP with GFP-beads. The results of the Co-IP assay further proved the interaction between 6K2 and ATG8f (Figure 3c). Finally, ATG8f and 6K2 were fused with GFP and RFP fluorescence tags, respectively, and were co-infiltrated into *N. benthamiana* leaves. Subcellular localization displayed that both green and red fluorescence signals were located at the edge of the cell membrane or cytoplasm, and the positions of the two fluorescence signals were mostly overlapping (Figure 3d). The above results confirmed that ATG8f interacted with ChiVMV-6K2 both in vitro and in vivo.

### 3.4. Silencing of ATG8f Promotes the Accumulation of ChiVMV-GFP

**Figure 4:**
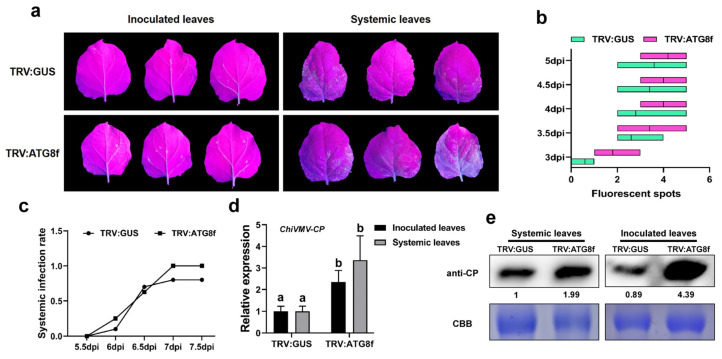
The silencing of *ATG8f* promotes the infection of ChiVMV-GFP. (**a**) Symptoms shown on inoculated and systemic leaves of the *ATG8f*-silenced plants (TRV: ATG8f) and the control plants (TRV: GUS) at 5 dpi. (**b**) The number of fluorescent spots on inoculated leaves at different time points. (**c**) The systemic infection rate of the virus at different time points after ChiVMV inoculation. (**d**) Relative expression of viral *CP* in inoculated and systemic leaves of ChiVMV-GFP-infected plants. (**e**) Accumulation of viral coat protein in inoculated and systemic leaves of ChiVMV-GFP-infected plants. Data are shown as means ± SD (*n* = 3). Lower case letters indicate statistically significant differences (*p* < 0.05).

It was reported in a previous study that the deficiency of ATG8a enhanced the accumulation of NIb and promoted the infection of TuMV [44]. In the present study, after confirming the direct interaction between 6K2 and ATG8f, the role of ATG8f in the autophagy-mediated plant antiviral process was still unknown. Thus, *ATG8f* was silenced by VIGS, and then the *ATG8f*-silenced plants were inoculated with ChiVMV-GFP (Figure 4a and Supplementary Figure S3a). The results showed that the number of virus fluorescent spots in virus-inoculated leaves was higher in *ATG8f*-silenced plants than that in the TRV: GUS-infiltrated plants (Figure 4b). The systemic infection rate in systemic leaves was also higher in *ATG8f*-silenced plants than that in the TRV: GUS-infiltrated plants at 5 dpi (Figure 4c). Accordingly, the expression of viral *CP* and the accumulation of viral coat protein in inoculated leaves or systemic leaves were higher in *ATG8f*-silenced plants than that in TRV: GUS-infiltrated plants (Figure 4d,e).

### 3.5. Overexpression of ATG8f Inhibits the Infection of ChiVMV-GFP

**Figure 5:**
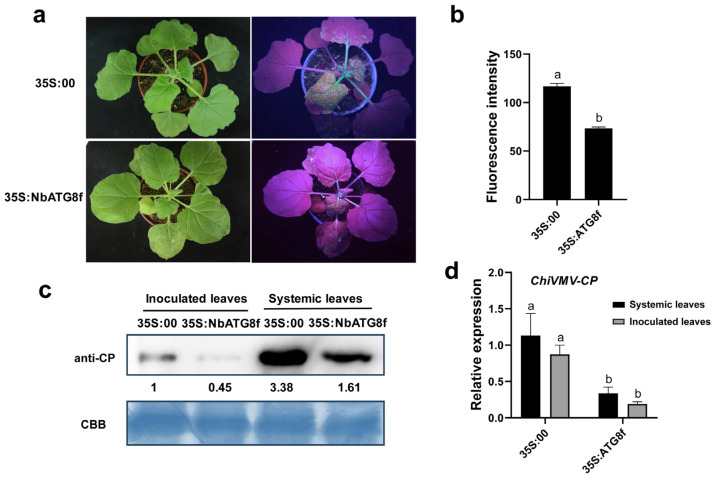
Overexpression of *ATG8f* inhibits the infection of ChiVMV-GFP. (**a**) Symptoms of systemic leaves in the *ATG8f*-overexpressing plants (35S: ATG8f) and the control plants (35S: 00) at 5 dpi. (**b**) Fluorescent intensity in systemic leaves at 5 dpi. (**c**) Accumulation of viral coat protein in inoculated and systemic leaves of ChiVMV-GFP-infected plants at 5 dpi. (**d**) Relative expression of viral *CP* in inoculated and systemic leaves of ChiVMV-GFP-infected plants. Data are shown as means ± SD (*n* = 3). Lower case letters indicate statistically significant differences (*p* < 0.05).

Next, plants with transient overexpression of *ATG8f* were used to explore the function of ATG8f in plants’ response to ChiVMV infection further. The results exhibited that there was lower virus fluorescence intensity in systemic leaves of *ATG8f*-overexpressing plants compared to that in the control plants (35S: 00) (Figure 5a,b and Supplementary Figure S3b,c). Corresponding to the symptoms and manifestations, the results of the qPCR and Western blot analysis showed that the accumulation of viral coat protein and the expression of viral *CP* were lower in *ATG8f*-overexpressing plants compared to that in the control plants (35S: 00) (Figure 5c,d). Combined with the results in Section 3.4 regarding the increased susceptibility in *ATG8f*-silenced plants, it could be concluded that *ATG8f* played a positive role in the antiviral response of *N. benthamiana* to the infection of ChiVMV-GFP.

### 3.6. 6K2 Is Degraded by Autophagy

**Figure 6:**
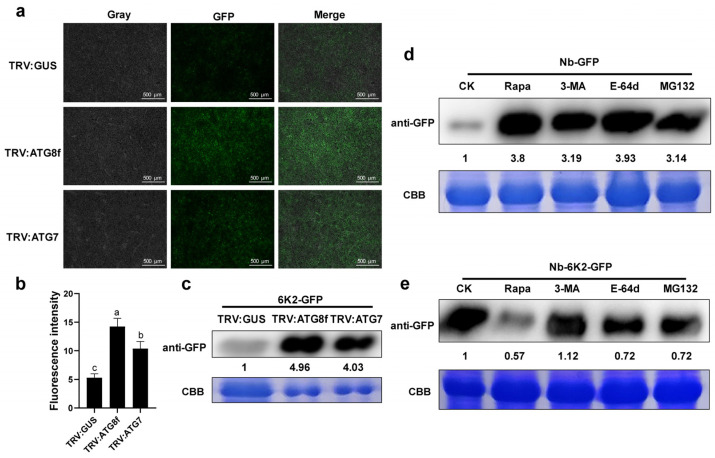
The 6K2-GFP is degraded through the autophagy pathway. (**a**) Fluorescence observation in *ATG7*- or *ATG8f*-silenced plants two days after infiltration with the 6K2-GFP vector. Bars = 500 μm. (**b**) Statistics of fluorescence intensity of 6K2-GFP in different test groups. (**c**) Protein accumulation of 6K2-GFP in the *ATG7*- or *ATG8f*-silenced plants and control plants (TRV: GUS). (**d**) Fluorescence intensity of GFP in *N. benthamiana* plants treated with Rapamycin, 3-MA, E-64d, and MG132, respectively. (**e**) Protein accumulation of 6K2-GFP in *N. benthamiana* plants treated with Rapa, 3-MA, E-64d, and MG132, respectively. Data are shown as means ± SD (*n* = 3). Lower case letters indicate statistically significant differences (*p* < 0.05).

It has been reported in previous studies that autophagy could target plant viral proteins through direct recognition by ATG8 [23,25]. Since, in the previous experiment, ATG8f was proved to be positively correlated with plants resistance, and there was a direct interaction between ATG8f and 6K2, we speculated that the degradation of 6K2 protein might also be associated with autophagy. Therefore, the 6K2 protein fused with GFP (6K2-GFP) was agroinfiltrated into *ATG7*- or *ATG8f*-silenced *N. benthamiana* plants to monitor the expression of 6K2-GFP. The results showed that stronger fluorescence was observed in *ATG7*- or *ATG8f*-silenced plants compared to that in TRV: GUS plants (Figure 6a,b), and the accumulation of 6K2-GFP fusion proteins in *ATG7*- or *ATG8f*-silenced plants was significantly higher than that in TRV: GUS plants (Figure 6c). To test whether the 6K2 protein was degraded by the autophagic or ubiquitin pathway, the 6K2-GFP-expressing plants were treated with an autophagy activator (Rapamycin), two autophagy inhibitors (3-MA and E-64d), and an inhibitor of the ubiquitin pathway (MG132), respectively; GFP-expressing plants were used as the control. The results showed that there were no significant fluctuations in the accumulation of GFP protein without 6K2 fusion under the treatments of Rapamycin, 3-MA, E-64d, and MG132, respectively (Figure 6d). However, the accumulation of GFP-6K2 was decreased in Rapamycin-treated plants and increased in those treated with E-64d or 3-MA (Figure 6e). Furthermore, the amount of 6K2-GFP protein in MG132-treated plants was similar to that in E-64d-treated plants. These results suggested that the degradation of 6K2-GFP protein might be independent of the ubiquitin degradation pathway, but mainly through the autophagy pathway.

## 4. Discussion

In this study, we showed that autophagy is involved in responses to ChiVMV infection in *N. benthamiana*. ChiVMV infection caused the accumulation of autophagosomes in infected *N. benthamiana* leaves and the upregulation of *ATGs*. Furthermore, ATG8f interacts with the ChiVMV 6K2 protein, promoting the degradation of 6K2 through the autophagy pathway.

Autophagy responds to the occurrence of factors that are detrimental to plant growth, including viral infection [18,23]. As the core adaptor of autophagosome, ATG8 has been widely reported to interact with plant endogenous proteins [22,45], but it is also a key interaction target of pathogens, including viruses, e.g., ATG8h interaction with *Geminivirus* nuclear protein C1 from tomato leaf curl Yunnan virus (TLCYnV) by a potential AIM motif to degraded C1 by autophagy [25]. NBR1, a selective autophagy receptor protein bonding with ATG8, interacted with P4 from *Caulimovirus* to mediate the degradation of P4-associated viral particles [46]. On the other hand, some studies demonstrated that autophagy can be manipulated or evaded by some viral factors. The γb protein from the barley strip mosaic virus (BSMV) directly bound itself to autophagy key regulator ATG7 to disrupt the interaction between ATG7 and ATG8, thereby repressing the formation of autophagosomes [44]. Hafrén et al. showed that TuMV appeared to antagonize NBR1-dependent selective autophagy by VPg and 6K2 during infection [33]. In the present study, the expression of ATG8f was correlated with the response of *N. benthamiana* to ChiVMV infection, and ATG8f played a positive role in the antiviral response of *N. benthamiana* to ChiVMV-GFP.

Furthermore, 6K2, a small integral membrane protein coded by potyvirus, could remodel the host ER for the formation of VRCs and contribute to viral replication [47,48]. In addition, 6K2-induced vesicles could also move from cell to cell during TuMV infection [49]. The 6K2 of the sugarcane mosaic virus could interact with lactate dehydrogenase to support virus infection [50]. In addition to its functions related to viral pathogenicity, TuMV 6K2 could serve as an elicitor for the unfolded protein response (UPR) and upregulate the selective autophagy receptor gene NBR1 in a UPR-dependent manner [27]. TuMV 6K2-derived VRCs are always co-localized with some autophagy proteins, like ATG8a, Beclin1, and NBR1 [24]. In the present study, ChiVMV 6K2 directly interacted with ATG8f, indicating that 6K2 protein might be targeted for degradation by autophagy.

Autophagy acts as a double-edged sword when fighting plant viruses: it can serve as a dependent pathway for plant antiviral activity and can also be favored by viruses [33,51,52,53]. For example, ATG8 interacted with movement protein (MP) from *Citrivirus* to mediate the degradation of MP, thus limiting viral movement [51]. NBR1 bonded with ATG8 to mediate the degradation of HCPro-associated PGs of TuMV [33]. On the contrary, TuMV VPg interacted with antiviral host factors and mediated their autophagic degradation to counteract Remorin-mediated and Suppressor of Gene Silencing 3 (SGS3)-mediated antiviral activities [52,53]. In the present study, the interaction between ATG8f and ChiVMV 6K2 was beneficial for plants. The interaction between ATG8f and ChiVMV 6K2 promoted the degradation of viral factor 6K2 through the autophagy pathway. Therefore, our results provided new evidence for the role of autophagy in the “arms race” between plants and viruses. However, it is not yet known whether other viral proteins encoded by ChiVMV can also be degraded by autophagy pathways. Future work is worthwhile to comprehensively explore the interaction between viral proteins and autophagy-related proteins and provide clues for developing potyvirus resistant cultivars.

## Data Availability

The raw data supporting the conclusions of this article will be made available by the authors, without undue reservation.

## Supplementary Materials

The following supporting information can be downloaded at https://www.mdpi.com/article/10.3390/v15122324/s1, Figure S1: Effects of silencing of autophagy-related genes on ChiVMV infection; Figure S2: Y2H screening of the interaction between ChiVMV-encoded proteins and autophagy-related proteins; Figure S3: Identification of the effects of silencing or overexpressing of *ATG8f* in *N. benthamiana* plants; Table S1: Primers used in the present study.
