# Supplementary material for: ATG8f Interacts with Chilli Veinal Mottle Virus 6K2 Protein to Limit Virus Infection
Source: Viruses. 2023 Nov 26;15(12):2324. doi: 10.3390/v15122324 (PMC10747504; doi:10.3390/v15122324)
Supplement: Supplementary file 1 [file viruses-15-02324-s001.zip › viruses-2740828-supplementary.pdf]

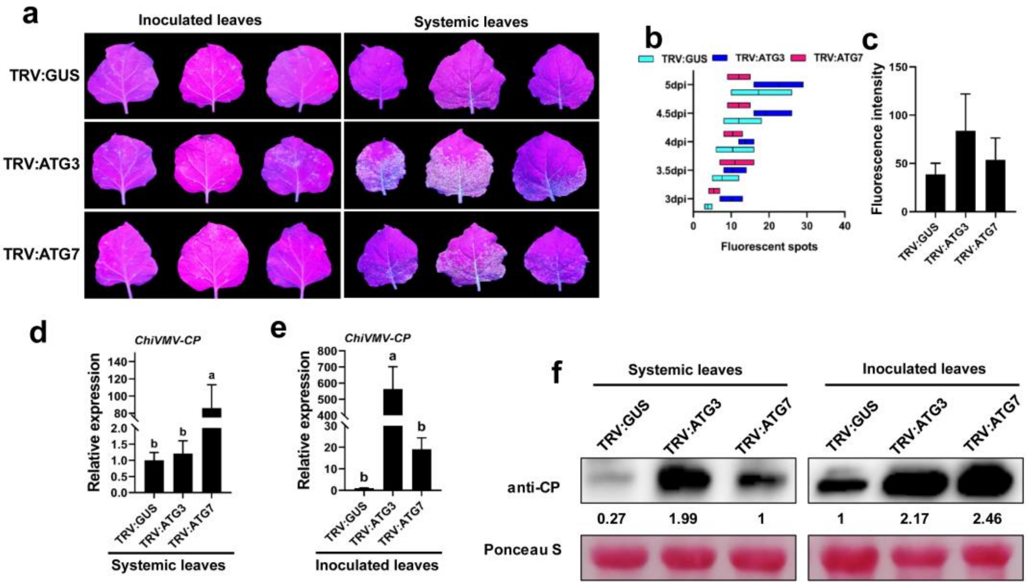

Figure S1. Effects of silencing of autophagy genes on ChiVMV infection. (a) Symptom on *ATG3* or *ATG7* silenced *N. benthamiana* plants inoculated with ChiVMV-GFP. (b) Fluorescent photographs of inoculated leaves. (c) Fluorescence intensity of GFP in systemic leaves. (d) Relative expression of viral *CP* in systemic leaves. (e). Relative expression of viral *CP* in ChiVMV-GFP inoculated leaves (f) Accumulation of viral coat protein in inoculated and systemic leaves. Significant differences were determined by one-way ANOVA (**P < 0.01, ***P < 0.001).

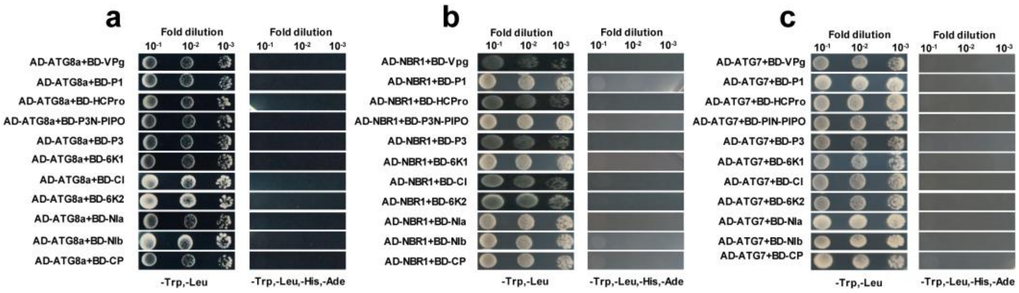

Figure S2. Y2H screening of the interaction between autophagy-related proteins and ChiVMV encoded proteins. (a) Screening of the interaction between NbATG8aand ChiVMV encoded proteins. (b) Screening of the interaction between NbNBR1 and ChiVMV encoded proteins. (c) Screening of the interaction between NbATG7 and ChiVMV encoded proteins.

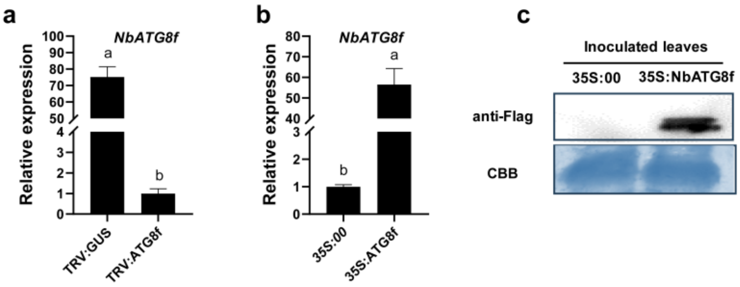

Figure S3. Identification of the effects of *NbATG8f* silencing or overexpressing in *N. benthamiana* plants. (a) qRT-PCR analysis of *NbATG8f* expression in control or silenced plants. (b) qRT-PCR analysis of *NbATG8f* expression in control or overexpressed plants. (c) Western blot analysis of NbATG8f expression in control or overexpressed plants. Significant differences were determined by one-way ANOVA (**P < 0.01, ***P < 0.001).

Table S1. Primers used in the present study.

Primer Names	Primer Sequences (5'-3')	Purpose
ChiVMV-CP-F	TCTTGGTATGAGGGTGTT	qPCR
ChiVMV-CP-R	GTCTAAATGATGGCTTGG	
NbActin-F	GATGAAGATACTCACAGAAAGA	
NbActin-R	GTGGTTTCATGAATGCCAGCA	
NbATG3-F	CGGCGATAATCTCGTCTC	
NbATG3-R	TGTCATCCTCGTCACTTT	
NbATG5-F	CCTCCTCTTCTAAATCACC	
NbATG5-R	TCATCCATTGGCATCAGT	
NbATG7-F	TGACTGTCTAAATGGTGGCA	
NbATG7-R	ACCGACTTTCCCGTGTAT	
NbATG8f-F	TTGAGAAGAGGCGTGCTG	
NbATG8f-R	CATCTGTTGGTGGTAGGA	
Nbbeclin1-F	AGAACTGCCACAATCCTC	
Nbbeclin1-R	TTCCATAGCCTTCCCAAA	
NbNBR1-F	TTGCGGGTATCTGTGAGG	
NbNBR1-R	AAGGCTGATTCTGGTAGTAGGA	
NbPI3K-F	CAGGAATCAGGAGCAAAC	
NbPI3K-R	AAGAAACTTAGTCAGAGCC	
TRV-ATG3-F	GGAATTCAAATGATGGCTGGCTT	VIGS
TRV-ATG3-R	CGGGGTACCTTATCATACGTGATGCTGA	
TRV-ATG7-F	GGAATTCATGGCGGATAGTGGA	
TRV-ATG7-R	CGGGATTCGTTGCAGGAGGATCAAG	
TRV-ATG8f-F	GAATTCATGGCAAAGAGTTCATTCAA	
TRV-ATG8f-R	CGGGATCCTTGCGAATGACATAG	
AD-ATG8a-F	GAATTCATGGCCAAAAGCTCCTTCA	Yeast two-hybrid
AD-ATG8a-R	GGATCCTTAGAACGATCCGAATGT	
AD-ATG8f-F	GAATTCATGGCAAAGAGTTCATTCAA	
AD-ATG8f-R	GGATCCTTACACCAAGTTAAAGT	
AD-NBR1-F	GAATTCATGGCCATGGAGTCTGCTATTGT	
AD-NBR1-R	GGATCCCTACTGCTCTCCAGCAATAAGA	
AD-Beclin1-F	GAATTCATGACGAAAAATAGCAGCA	
AD-Beclin1-R	GGATCCTCAAGATTGAAACTTGGTATTAG	
AD-ATG7-F	GGAATTCATGGCGGATAGTGGA	
AD-ATG7-R	CCGCTCGAGTTATATTTCTATAGAGTCATC	
BD-VPg-F	CGCGGATCCATGGCACAAAAGAAGAACA	
BD-VPg-R	CGCGTCGACTTCATGCTCAACCCCTTCTC	
BD-P1-F	CGGAATTCATGGCTGTGCAAGTTACC	
BD-P1-R	CGGGATCCATAATGATTGACTTTAGGAAT	
BD-HCPro-F	CGCGGATCCATGTCAGCAGGTGAGCTC	
BD-HCPro-R	CGCGTCGACACCAACTCTGTACATCTTC	

BD-P3N-PIPO-F	CGGAATTCATGGGTGAAGGAATTGAAA	
BD-P3N-PIPO-R	CGGGATCCTTTTTCCATAATATGCAAGGAT	
BD-P3-F	CGGAATTCATGGGTGAAGGAATTGAAA	
BD-P3-R	CGGGATCCTTGATGACCCACTGGTTCCT	
BD-6K1-F	CGGAATTCATGGCAAGCAAAAGACCATC	
BD-6K1-R	CGGGATCCCTGGTGATTCACAGCTTC	
BD-CI-F	CGGAATTCATGAGCCTGGATACCATCTTAG	
BD-CI-R	CGGGATCCTTGATGCATGACTGTTTCCA	
BD-NIa-F	CGCGGATCCATGGCCCGCTCATTGAAC	
BD-NIa-R	CGCGTCGACCTGTTCACAAACCAGTG	
BD-NIb-F	TCCCCCGGGATGGCGAGCGAGAAGTGGTT	
BD-NIb-R	CGCGTCGACTTGGTGAATAACTTCAC	
BD-CP-F	CGCGGATCCATGGCAGGAGAGAGTGTTG	
BD-CP-F	CGCGTCGACTAATCCCCGAACGCCTAGC	
BD-6K2-F	CGGAATTCATGAGTATAGATGATATCAGCA	
BD-6K2-R	CGGGATCCTTGGTGGTTGACTTGTTCAC	
ATG8f-GFP-F	CGGGATCCATGGCAAAGAGTTCATTC	
ATG8f-GFP-R	ACGCGTCGACTTACACCAAGTTAAAGTCCC	
ATG8f-flag-F	CGGGATCCATGGCAAAGAGTTCATTC	
ATG8f-flag-R	ACGCGTCGACTTACACCAAGTTAAAGTCCC	Transient expression
6K2-GFP-F	CGGGATCCATGAGTATAGATGATATCAG	
6K2-GFP-R	ACGCGTCGACTTGGTGGTTGACTTGTTCAC	
6K2-RFP-F	ACGCGTCGACATGAGTATAGATGATATCAG	
6K2-RFP-R	CGGGATCCTTGGTGGTTGACTTGTTCAC	
nYFP-ATG8f-F	CGGGATCCATGGCAAAGAGTTCATTC	
nYFP-ATG8f-R	ACGCGTCGACCACCAAGTTAAAGTCCC	BiFC
cYFP-6K2-F	CAATTACAGGTACCCGGGGATCCAGTATAGATGATATC	
cYFP-6K2-R	CACGCTGCCACCGCCGTCGACATTGGTGGTTGACTTGT	
